# The complete mitochondrial genome of *Monochamus dubius* Gahan (Coleoptera: Cerambycidae)

**DOI:** 10.1080/23802359.2021.1882909

**Published:** 2021-03-01

**Authors:** Jiankai Wu, Ren Chen, Yi Wan, Shaozhen Wang, Jiayi Ma

**Affiliations:** a Fujian Provincial Forestry Science and Technology Extension Center, Fuzhou, China; b Comprehensive Technology Center of Putian Customs, Putian, China; c College of Forestry, Fujian Agriculture and Forestry University, Fuzhou, China

**Keywords:** Complete mitochondrial genome, *Monochamus dubius* Gahan, Chrysomeloidea, phylogenetic analysis

## Abstract

In this study, we sequenced the complete mitochondrial genome of *Monochamus dubius* Gahan 1894. The results showed that the length of complete mitochondrial genome was 16,029 bp with 22.22% GC content, containing 39.4% A, 38.4% T, 13.4% C, 8.8% G. There were 13 protein-coding genes (PCGs), 22 transfer RNA genes (tRNA), two ribosomal RNA genes (rRNA) and one AT-rich region. Phylogenetic analysis showed that *M. dubius* was clustered with *Monochamus urussovii* and *Monochamus alternatus alternatus,* and confirmed the sister relationship among the genus *Monochamus*, *Anoplophora*, and *Aristobia* from Cerambycidae. The complete mitogenome of *M. dubius* would help understand the classification and phylogeny of Chrysomeloidea.

Chrysomeloidea cover the two largest families, Cerambycidae (longhorn beetles) and Chrysomelidae (leaf beetles), each with more than 30,000 species and about a dozen subfamilies (Nie et al. 2020). Relationships among subfamilies of Chrysomelidae have been clarified over the past two decades (Reid 2000; Ge et al. 2011). However, up to date, phylogenetic relationships within Cerambycidae still remain understudied (Haddad et al. 2018). In recent decades, insect mitochondrial genome has been widely used in phylogenetic and population genetic studies at different levels due to its unique features (Salvato et al. 2008). Therefore, this study newly determined the complete mitochondrial genome of *M. dubius* from Cerambycidae. In addition, we built the phylogenetic tree based on the maximum likelihood method to understand the evolution relationship. The results provide important information for studying the evolution of mitochondrial genomes in this group.

The *M. dubius* adults were collected from Lianjiang, Fujian Province, China (East longitude 119.8002, North latitude 26.3284) by using sex pheromone traps. The specimens were deposited at −80 °C in the Key Laboratory of Integrated Pest Management in Ecological Forests, Fujian Agriculture and Forestry University (Jiankai Wu, forestry2020@126.com) under the voucher number TN-202009. The total DNA was extracted from the legs using Insect DNA Kit (Omega Bio-Tek, GA, USA) and purified using QIAquick Gel Extraction Kit (Qiagen GmbH, Germany). The mitochondrial genome was sequenced by the Illumina Hiseq 2500 (Illumina, CA, USA) at the Novogene (Beijing, China). A total of 66,458,876 clean reads were obtained by quality control and filtration from the 68,684,628 raw reads. After the de novo assembly of MitoZ and metaSpades software (Nurk et al. 2017), we obtained a 16,029 bp length complete mitochondrial genome of *M. dubius* with 22.22% GC content.

The characteristics of PCGs, tRNAs, rRNAs, and AT-rich region were obtained from the annotated result of mitoMaker (Bernt et al. 2013). The complete mitogenome sequence has been submitted to NCBI Genbank with accession number MW067124. In this study, 37 genes were annotated, including 13 PCGs, 22 tRNAs, 2 rRNAs. Thirteen PCGs are 10,911 bp in total, encoding 3,637 amino acids. Nine PCGs (ATP6, ATP8, COX1, COX2, COX3, CYTB, ND2, ND3, ND6) are clockwise coding, while four PCGs (ND1, ND4, ND4L, ND5) are counterclockwise coding. All PCGs stop with usual codons such as TAA and TAG, except ND5 which is stopped with the incomplete condon T. The *rrnS* and *rrnL* genes are 810 bp and 1234 bp in length.

To investigate its taxonomic status of *M. dubius*, the sequence alignment was performed with the MAFFT, according to mitochondrial genome sequence of 17 species from Chrysomeloidea which had been selected from Genebank BLAST according to the percent identity (Katoh and Standley 2013). Take the whole mitogenome of *Cnidocampa flavescens* (GenBank: KY628213.1) as an out-group, a maximum likelihood analysis of 11 Cerambycidae species and 6 Chrysomelidae species was performed with the software MEGA 7.0, following by calculating the bootstrap values with 1000 replications (Kumar et al. 2016). The resultant ML trees distinctly showed that the *M. dubius* constituted a monophyletic group with other 10 Cerambycidae. The monophyletic Cerambycidae that contains Lamiinae, Aseminae, Spondylinae and Lepturinae was assigned to the sister group to the clade of Chrysomelidae that consists of Chrysomelinae and Galerucinae in this study. Additionally, *M. dubius* was clustered together with *M. urussovii* and *M. alternatus alternatus* (Figure 1). The complete mitochondrial genome of *M. dubius* will provide useful genetic information for the genetic evolution of *M. dubius*, as well as in other insects of Chrysomeloidea.

**Figure 1.**
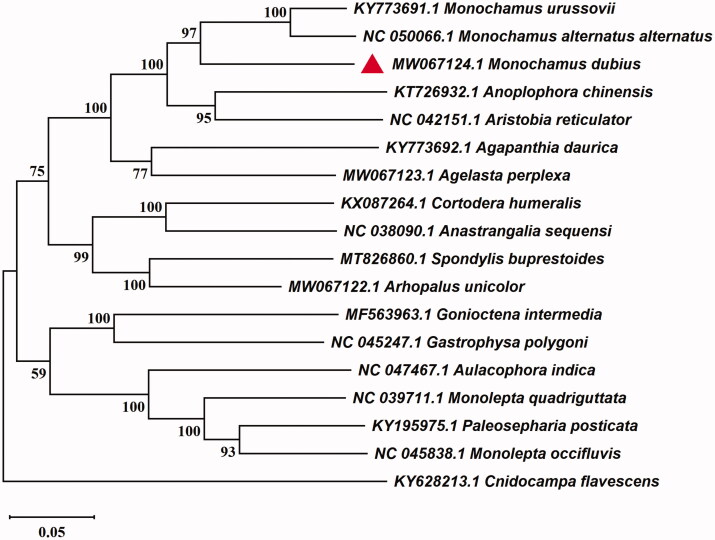
Maximum likelihood tree of the *Monochamus dubius, Cnidocampa flavescens,* and other 16 species of Chrysomeloidea based on the genome sequence. Numbers labeled on the branch are bootstrap values.

## Data Availability

The genome sequence data that support the findings of this study are openly available in GenBank of NCBI at [https://www.ncbi.nlm.nih.gov] (https://www.ncbi.nlm.nih.gov/nuccore/MW067124.1/) under the accession no. MW067124. The associated BioProject, SRA, and Bio-Sample numbers are PRJNA688627, SRP300485, and SAMN17183230 respectively.
